# Toxicity of the pharmaceuticals finasteride and melengestrol acetate to benthic invertebrates

**DOI:** 10.1007/s11356-020-10121-7

**Published:** 2020-07-22

**Authors:** Ève A. M. Gilroy, Adrienne J. Bartlett, Patricia L. Gillis, Nicholas A. Bendo, Joseph Salerno, Amanda M. Hedges, Lisa R. Brown, Emily A. M. Holman, Naomi L. Stock, Shane R. de Solla

**Affiliations:** 1 Green House Science, Burlington, ON Canada; 2 Aquatic Contaminant Research Division, Environment and Climate Change Canada, Burlington, ON L7S 1A1 Canada; 3 Ecotoxicology and Wildlife Health Division, Environment and Climate Change Canada, Burlington, ON Canada; 4 Water Quality Centre, Trent University, Peterborough, ON Canada

**Keywords:** Freshwater mussels, Amphipods, Mayflies, Reproduction, Growth, Pharmaceuticals, ECOSAR

## Abstract

The toxicity of endocrinologically active pharmaceuticals finasteride (FIN) and melengestrol acetate (MGA) was assessed in freshwater mussels, including acute (48 h) aqueous tests with glochidia from *Lampsilis siliquoidea*, sub-chronic (14 days) sediment tests with gravid female *Lampsilis fasciola*, and chronic (28 days) sediment tests with juvenile *L. siliquoidea*, and in chronic (42 days) sediment tests with the amphipod *Hyalella azteca* and the mayfly *Hexagenia* spp. Finasteride was not toxic in acute aqueous tests with *L. siliquoidea* glochidia (up to 23 mg/L), whereas significant toxicity to survival and burial ability was detected in chronic sediment tests with juvenile *L. siliquoidea* (chronic value (ChV, the geometric mean of LOEC and NOEC) = 58 mg/kg (1 mg/L)). Amphipods (survival, growth, reproduction, and sex ratio) and mayflies (growth) were similarly sensitive (ChV = 58 mg/kg (1 mg/L)). Melengestrol acetate was acutely toxic to *L. siliquoidea* glochidia at 4 mg/L in aqueous tests; in sediment tests, mayflies were the most sensitive species, with significant growth effects observed at 37 mg/kg (0.25 mg/L) (ChV = 21 mg/kg (0.1 mg/L)). Exposure to sublethal concentrations of FIN and MGA had no effect on the (luring and filtering) behaviour of gravid *L. fasciola*, or the viability of their brooding glochidia. Based on the limited number of measured environmental concentrations of both chemicals, and their projected concentrations, no direct effects are expected by these compounds individually on the invertebrates tested. However, organisms are exposed to contaminant mixtures in the aquatic environment, and thus, the effects of FIN and MGA as components of these mixtures require further investigation.

**Electronic supplementary material:**

The online version of this article (10.1007/s11356-020-10121-7) contains supplementary material, which is available to authorized users.

## Introduction

Over the last two decades, it has been increasingly clear that pharmaceutical and personal care products (PPCPs) are ubiquitous in environmental surface waters near populated areas. Pharmaceuticals are designed to be biologically active and affect specific processes in target organisms, and concerns regarding the potential to incur effects on non-target species have generated considerable research on this topic (López-Pacheco et al. 2019). Numerous pharmaceuticals have been detected in surface waters at concentrations ranging between ng/L and μg/L, indicating a substantial potential for exposure. What remains unclear is the extent to which pharmaceuticals pose a hazard to aquatic organisms.

Municipal wastewater effluent discharges and land application of treated biosolids are significant sources of PPCPs to aquatic and terrestrial environments. Effluents from Canadian municipal wastewater treatment plants contain numerous PPCPs (e.g., Servos et al. 2005; Lajeunesse et al. 2012; Muir et al. 2017), which are continually released into aquatic ecosystems, occasionally at concentrations that could cause effects to aquatic organisms. Approximately 10% of pharmaceuticals assessed in Europe, including hormones, antibiotics, analgesics, antidepressants, and antineoplastics, were estimated to pose environmental risks, largely (though not exclusively) to aquatic organisms (Küster and Adler 2014).

Recent studies have demonstrated that exposure to municipal wastewater effluents can impair populations of aquatic organisms. Effects in fish include disruption of immune competence, feminization of male fish, reduced fertilization success and egg survival, and increased incidence of intersex (Hébert et al. 2008; Tetreault et al. 2011; Bahamonde et al. 2015; Fuzzen et al. 2015; Lacaze et al. 2017). In addition, reduced immunocompetence, decreased lifespan, and condition factor have been documented in freshwater mussels exposed to municipal water effluents (Blaise et al. 2002; Gillis 2012; Gillis et al. 2014), and Gillis et al. (2017) reported an extirpation zone for freshwater mussel populations 7.5 km downstream of a large municipal wastewater treatment plant. Decreased reproduction of freshwater snails has also been observed downstream of a sewage treatment facility (Gust et al. 2014).

Concentrations of individual PPCPs in surface waters are frequently below those associated with direct toxicity to aquatic organisms (Boxall et al. 2012; Kostich et al. 2014; aus der Beek et al. 2016); however, PPCPs can reach toxic concentrations in surface waters downstream of discharges (e.g., Sanchez et al. 2011) and can bioaccumulate in some species, such as fish (Muir et al. 2017), freshwater snails (Gust et al. 2014), and freshwater mussels (de Solla et al. 2016).

In many cases, feminizing effects observed in fish exposed to wastewater effluent have been attributed to exposure to environmental estrogens. The synthetic hormone 17α-ethinylestradiol was demonstrated to have caused the collapse of a fish population at concentrations detected in the environment (Kidd et al. 2007). Hicks et al. (2017) reported decreased incidence of intersex in a population of rainbow darters downstream of a municipal sewage treatment facility, after plant upgrades, with corresponding decreases in the concentration of estrogens detected in the effluent. However, observed effects in invertebrates have not been as clear-cut, and questions remain as to whether these compounds have any hormonal action in molluscs (Scott 2012, 2013). Studies with other aquatic invertebrates have reported effects occurring at concentrations several orders of magnitude greater than those observed in aquatic systems (e.g., Dussault et al. 2008; Watts et al. 2001; Hutchinson et al. 1999), and at concentrations unlikely to be mediated via an endocrine response. Similarly, studies on the toxicity of antiandrogenic compounds report effects in aquatic invertebrates at concentrations greater than those reported to incur effects in aquatic vertebrates (Lalone et al. 2013; Yamamoto et al. 2011 and citations therein). For example, 21-day exposure to spironolactone reduced the fecundity of Japanese medaka (*Oryzias latipes*) and fathead minnow (*Pimephales promelas*) at a concentration of 50 μg/L, whereas 21-day exposure to as much as 500 μg/L had no effect on the reproduction of *Daphnia magna* (Lalone et al. 2013). Exposure to seven antiandrogenic parabens induced the synthesis of vitellogenin in medaka in 14-day exposures (NOECs 20–160 μg/L), but affected the mobility of *D. magna* at concentrations at least an order of magnitude greater (21-day NOECs of 640 μg/L to 2400 μg/L; Yamamoto et al. 2011, and citations therein).

The present study aimed to assess the toxicity of two synthetic hormones, finasteride and melengestrol acetate, to benthic organisms in aqueous and sediment tests. These PPCPs were selected due to the paucity of data regarding potential effects on non-target organisms. They have similarly high log K_OC_ and log K_OW_ values, and thus have a tendency to bind to soils and sediments and to bioaccumulate (Table 1). Finasteride (FIN) is an antiandrogen used to treat hyperplasia of the prostate and male pattern baldness through the inhibition of steroid 5α-reductase type 2, an enzyme which converts testosterone to 5α-dihydrotestosterone (reviewed in Langlois et al. 2010). Melengestrol acetate (MGA) is a steroidal progestin (synthetic progesterone) used as a feed additive to promote growth in cattle and is an androgen antagonist and antigonadotropin. Synthetic hormones are frequently given to cattle, either as additives in feedstock or as subcutaneous implants, and can enter the aquatic environment through surface runoff or via airborne deposition (Sandoz et al. 2018).

**Table 1:**
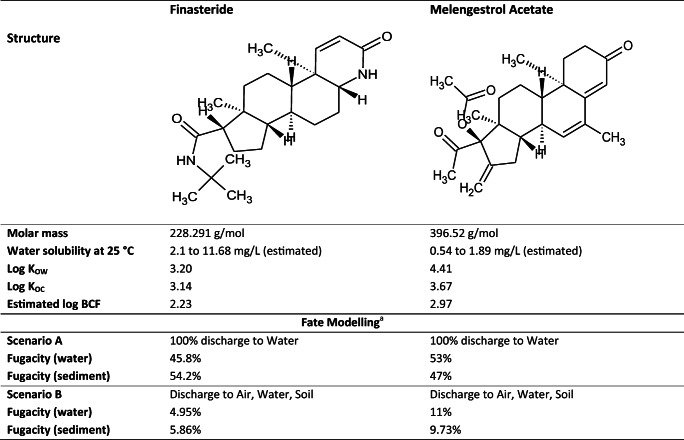
Structure and chemical properties of finasteride and melengestrol acetate, based on ACD/Percepta (ACD/Labs 2015) and Epi Suite (US EPA 2013). Fugacity estimates were based on discharges solely to water, and equally divided between air, soil, and water

^a^The fate of finasteride and melengestrol acetate within aquatic systems was assessed using level III multimedia fugacity models through US EPA’s Episuite (US EPA 2013)

There are few measurements of FIN or MGA in surface waters or environmental exposure estimates. Howard and Muir (2011) identified FIN as a potentially persistent and bioaccumulative high production volume pharmaceutical, and Lindim et al. (2019) measured concentrations of approximately 0.05 ng/L in surface waters in Sweden. Treated solid manure contained 0.3 to 8 μg/kg MGA (Schiffer et al. 2001), and Shen et al. (2018) reported concentrations of 0.04 to 0.33 ng/L MGA in the River Wenyu and its tributaries in Beijing, China. Bartelt-Hunt et al. (2012) measured MGA in runoff from an animal research facility designed to mimic a commercial cattle production facility, and detected MGA in 6% of the runoff samples from treated cattle, the maximum concentration being 115 ng/L.

The objectives of the present study were to assess the toxicity of FIN and MGA to four species of benthic invertebrates (two mussel species, amphipods and mayflies) using laboratory exposures, and to compare the data generated in the present study to toxicity data obtained using quantitative structure-activity models (QSARs). Benthic organisms were the focus of the present study, as they would be exposed to PPCPs through both sediment and aqueous routes in the environment. A series of toxicity tests was conducted to assess the toxicity of FIN and MGA: (i) acute (aqueous) and chronic (sediment) toxicity tests using the freshwater mussels *Lampsilis siliquoidea* and *Lampsilis fasciola* (viability/survival, behaviour, algal clearance rate), (ii) chronic sediment tests using the amphipod *Hyalella azteca* (survival, growth, sex ratio, and reproduction), and (iii) chronic sediment tests using the mayfly *Hexagenia* spp. (survival, growth).

## Materials and methods

### Chemicals

Finasteride (C_23_H_36_N_2_O, CAS 98319-26-7) and melengestrol acetate (C_25_H_32_O_4_, CAS 2919-66-6) were obtained from Sigma Aldrich (Oakville, ON). Deuterium-labelled FIN (FIN-D_9_, C_23_H_27_D_9_N_2_O) was purchased from Santa Cruz Biotechnology Inc. (Dallas, TX), and deuterium-labelled MGA (MGA-D_10_, C_25_H_22_D_10_O_4_) was obtained from CDN Isotopes (Pointe-Claire, QC). All standards were stored at 4 °C and used as received. Stock solutions (1000 mg/L) in methanol (HPLC grade; Fisher Scientific, Whitby, ON) were prepared for standards used in chemical analysis. Stock solutions for spiking of water and sediments were prepared in acetone (HPLC grade; Fisher Scientific, Whitby, ON) and refrigerated until use.

### Sediment preparation

Sediments commonly used by Environment and Climate Change Canada (ECCC) as reference sediments for toxicity testing and invertebrate culturing were collected from two locations in Lake Erie (Long Point Bay and Long Point Marsh; ON, Canada) and stored at 4 °C until preparation. The sediments were sieved separately (500 μm) and then combined to achieve a composition of approximately 2% organic matter and a sediment density of 1.5 g/mL (Prosser et al. 2017a).

Finasteride and MGA were spiked individually into the sediments in 1-L amber glass jars. Nominal concentrations were 10, 30, 100, 300, and 1000 mg/kg for juvenile freshwater mussels and 3, 10, 30, 100, and 300 mg/kg for amphipods and mayflies, and were based on the results of range-finding tests (detailed in Supplementary Materials). Negative control (no acetone or PPCP added) and solvent control sediments (2% (FIN) and 1.25% (MGA) acetone, selected for their relative solubility in that solvent) were also prepared. Testing with gravid female mussels included a negative control, a solvent control for each compound (2% (FIN) and 1.25% (MGA)), and a single sublethal treatment of 10 mg/kg FIN or 100 mg/kg MGA (nominal). For each PPCP, the concentration of solvent used was consistent across treatments.

The sediments were mixed for 24 h, left open in a fume hood for 3–5 days for the acetone to evaporate, and then stored at 4 °C until use. Seven to ten days after spiking, 100 mL of sediment was transferred to 1-L beakers with 750 mL of culture water (dechlorinated Burlington, ON city tap water, pH 8.3 ± 0.03, dissolved oxygen 8.2 ± 0.35 mg/L, conductivity 346 ± 40 μS/cm, ammonia not detected, dissolved organic carbon 1.9 ± 0.65 mg/L, alkalinity 89 ± 0.9 mg/L, hardness 128 ± 3.7 mg/L, calcium 36 ± 1.1 mg/L, chloride 29 ± 4.7 mg/L, magnesium 9 ± 0.2 mg/L, potassium 1.7 ± 0.09 mg/L, sodium 17 ± 3.1 mg/L; test-specific water quality parameters are provided in Table S1) for toxicity testing with freshwater mussels and mayflies, and 50 mL of sediment was transferred to 600 mL beakers with 375 mL of culture water for testing with amphipods. All beakers were aerated for 1 week under testing conditions prior to the addition of organisms, and throughout the tests. Duration of toxicity tests varied depending on the species and endpoint of interest (see below). Water quality parameters were collected for each test (Table S1).

### Toxicity testing

#### Freshwater mussels

Freshwater mussels can be exposed to contaminants dissolved in water, sequestered in surficial sediment, or bound to suspended particulates, and therefore, the toxicity of FIN and MGA was determined using three mussel life stages that occupy different habitats. Glochidia (larval stage) from *Lampsilis siliquoidea* were used to determine acute toxicity in 48 h aqueous tests, and spiked sediment tests with juvenile *L. siliquoidea* were conducted to assess the chronic (28 days) effect of sediment exposure. Sub-chronic (14 days) sediment tests were conducted with gravid (adult) female *Lampsilis fasciola* to assess behaviour and effects on the viability of brooding glochidia. Gravid *L. siliquoidea* were not available at the time of experimentation, so the closely related *L. fasciola* was selected for sub-chronic testing.

##### Glochidia

Aqueous acute toxicity tests were based on the American Society for Testing and Materials (ASTM 2013) protocols for conducting toxicity tests with early life stages of freshwater mussels. Gravid female *L. siliquoidea* were collected from an established reference site in the Maitland River (ON) and kept in ECCC’s Aquatic Life Research Facility (Canada Centre for Inland Waters, Burlington, ON). Glochidia were removed from gravid females and their viability (i.e., ability to close their valves) was assessed. Glochidia from a minimum of three gravid mussels whose glochidia exceeded 90% viability were pooled and exposed to a geometric series of nominal concentrations (0, 0.0025, 0.025, 0.25, 2.5, 25 mg/L) of either FIN or MGA plus a solvent control. Tests were conducted in 250-mL glass beakers (unaerated and unfed) with culture water. After 24 and 48 h of exposure, glochidia viability was assessed in a minimum of 100 glochidia per replicate (four replicates). Water samples were collected at the beginning and end of the tests, and frozen pending chemical analysis.

##### Juvenile mussels

Freshwater mussels (*L. siliquoidea*, 6–8 months old, ~ 1 cm in length) were purchased from Missouri State University (Springfield, MO, USA). Mussels had been reared in the laboratory and fed live algae (*Neochloris oleoabundans*) for the first 6 months (Barnhart 2006).

Ten juvenile mussels were added to each beaker containing culture water and spiked sediment (five replicates per concentration), and fed 200 μL of an algae mixture (4.5·10^8^ cells Nanno 3600—*Nannochloropsis*) and 0.5·10^8^ cells Shellfish Diet 1800 (Reed Mariculture Inc., Campbell, CA; Gilroy et al. 2014) twice daily on weekdays, and daily on weekends. After 28 days, mussels were recovered and the survivors were counted and transferred to Petri dishes for 24 h to assess burial ability (Prosser et al. 2017b). Mussel behaviour was observed daily for 72 h, and those that were widely gaping or showed no foot movement were considered dead.

##### Gravid female mussels

Gravid female *L. fasciola* were collected from a reference site in the Speed River (ON) and maintained in the Aquatic Life Research Facility. A 14-day exposure was conducted at concentrations that were sublethal to juvenile mussels in range-finding tests (Table S2) to determine if exposure to FIN or MGA would elicit effects on filtering activity, lure display, and viability of the brooding glochidia. The behaviour of gravid females was observed for 2 days prior to testing, and those displaying lures were selected for the experiment. The viability of glochidia from each luring female was assessed and mussels with glochidia exceeding 80% viability were used in the exposure. Each gravid mussel was transferred to one of five replicate 1-L beakers containing culture water and either control sediment, solvent control (2% acetone [FIN] or 1.25% acetone [MGA]), and 10 mg/kg FIN or 100 mg/kg MGA (nominal). Each replicate was fed 1 mL of an algae mixture (see above) twice daily on weekdays. Behaviour (filtering activity [i.e., visibly open siphons] and lure display) was recorded three times daily on weekdays. The mussels were removed from their treatments after 14 days, and the viability of glochidia from each female was reassessed.

#### Amphipods

Amphipods (*Hyalella azteca*) were cultured as described by Borgmann et al. (1989). Dechlorinated municipal tap water was used for cultures and toxicity tests. Both cultures and toxicity tests were held at 25 °C with a photoperiod of 16 h light:8 h dark, and amphipods were fed finely ground Tetra-Min fish food flakes (Tetra GMBH, Melle, Germany). Juvenile amphipods were removed weekly from breeding containers and used in toxicity tests.

Chronic sediment tests (42 days, static) with *H. azteca* were conducted. Twenty juvenile amphipods (3–11 days old) were added to each beaker (five replicates for each control and PPCP concentration). Two tests were conducted for each compound. Amphipods were fed ground Tetra-Min twice per week during weeks 1 and 2, three times during week 3, and 5 mg three times per week during weeks 4 to 6. Surviving adults were counted, weighed as a group, and examined under a dissecting microscope to identify males (enlarged second gnathopods) and females, and the number of juveniles was recorded.

#### Mayflies

Mayfly collection and culturing methods are described in detail elsewhere (Hanes and Ciborowski 1992; Bartlett et al. 2018). Briefly, eggs of *Hexagenia* spp. (mixture of *H. rigida* and *H. limbata*) were collected from gravid females in June 2015 and stored at 4 °C. Prior to testing, eggs were hatched and nymphs were grown in aerated 20-L aquaria containing culture sediment (depth: 2.5 cm) and culture water (depth: 10 cm) for 6 to 7 weeks.

Chronic sediment tests (42 days, static) were conducted with *Hexagenia* spp. Ten mayflies (5–8 mg wet weight) were added to each beaker (three replicates for each control and PPCP concentration). Two tests were conducted for each compound. Mayflies were fed 50 mg of a mixture of cereal wheat grass, Brewer’s yeast, and ground Tetra-min per beaker per week (Bartlett et al. 2018). At the end of the test, mayflies were removed and surviving animals from each of the three replicates were counted and weighed.

### Chemical analysis

Samples for chemical analysis were collected from each test completed for each species. One water and one sediment sample (when sediment was present) were collected from each control and PPCP treatment at the beginning (prior to the addition of organisms) and end of the exposure (either as composite samples or as sub-samples from one replicate, depending on feasibility of collection for each species). All samples were frozen at − 20 °C, freeze-dried, and shipped to the Water Quality Centre, Trent University (Peterborough, ON), for analysis. Water samples were filtered using glass microfiber filters (1.2 μm) under vacuum. Those with expected concentrations > 10 ppb were diluted 1:1 with methanol, while those with trace levels (< 10 ppb) were concentrated using solid phase extraction with Oasis HLB cartridges (Waters, Mississauga, ON), and eluted using 20:80 methanol:acetone (HPLC grade; Fisher Scientific, Whitby, ON). Extracts were evaporated to dryness with a stream of nitrogen and reconstituted in 200 μL of 50:50 methanol:water. Sediment samples were centrifuged to remove pore water, freeze-dried, and homogenized. Freeze-dried sediments (1 g) were extracted in polypropylene centrifuge tubes using 5 mL of methanol and sonicated for 10 min. The extracts were then centrifuged and the solvent layers transferred to a clean centrifuge tube. The extraction procedure was repeated twice and the solvent layers (~ 15 mL total) were combined. Sample volume was reduced to 10 mL under a nitrogen stream. Samples were analysed using a Shimadzu 10A liquid chromatography instrument and a Perkin Elmer 200 Series autosampler paired with an AB Sciex Qtrap 5500 mass spectrometer (Concord, ON), operated in positive ionization mode. Further details on chemical analysis are included in the Supplemental Material. All reported values are based on measured concentrations. All toxicity data from sediment tests are presented as measured concentrations in sediment (mg/kg dry weight), followed by measured concentrations in overlying water in parentheses (mg/L).

### Modelling

#### Fugacity

Given the paucity of data on the distribution of FIN and MGA in the environment, their fate within aquatic systems was assessed using level III multimedia fugacity models through US EPA’s Episuite (US EPA 2013), to gain a better understanding of their behaviour in aquatic environments and under the test conditions of the current study.

#### Toxicity estimation

As there are presently few data available on the toxicity of FIN and MGA in non-target aquatic organisms, expected toxicities were estimated using the Organic Module of US EPA’s ECOlogical Structure-Activity Relationship Model (ECOSAR) application version 2.0 (US EPA 2017). The estimates based on the various chemical classes found in the structure of each compound were noted, and the most conservative value was retained for comparison with the data generated by the laboratory toxicity tests.

### Statistical analyses

Differences in survival and growth among treatments were tested using analysis of variance (ANOVA). When the ANOVAs revealed significant values (*α* < 0.05), Tukey’s honestly significant difference post hoc tests were performed to identify treatments that differed significantly from controls. If no differences between the control and solvent control were detected, the two controls were pooled; if differences existed, comparisons were made to the solvent control (Green 2014). In case of violation of the assumptions of normal distribution of residuals and homoscedasticity, differences were tested using the Kruskal-Wallis test, followed by Mann-Whitney *U* tests to determine differences from controls if the Kruskal-Wallis test was significant (*α* < 0.05). The data were not amenable to calculation of ECx, as the differences in observed effects between controls and PPCP concentrations were less than 50% or few partial effects were obtained. Therefore, lowest-observed effect concentrations (LOECs) and no-observed effect concentrations (NOECs) were identified, and chronic values (ChV), the geometric means of the LOEC and NOEC, were calculated.

## Results

### Toxicity testing

#### Freshwater mussels

##### Glochidia

Glochidia viability (i.e., survival) was similar in the control (91 ± 1.1%, mean ± standard deviation) and the solvent control (89 ± 0.7%) treatments in the FIN and MGA exposure (Table 2) and therefore all control data were pooled. For both FIN (85 ± 1.9% at 23 mg/L; Table 2) and MGA (70 ± 1.4% at 4 mg/L; Table 2), viability was significantly reduced at the highest concentrations tested; however for FIN, this observed difference was less than a 10% change from the pooled control and therefore was not biologically meaningful. Viability in the MGA test was also significantly reduced (82%) at 0.05 and 1.9 mg/L compared with the pooled control, but not at 0.3 mg/L; all were still within 10% of controls (Table 2).

**Table 2:** Viability (mean, % (standard deviation), *n* = 4) of *Lampsilis siliquoidea* glochidia after a 48-h exposure to finasteride or melengestrol acetate (mean measured concentrations^a^; mg/L). Effect data in bold are significantly different from controls

			**Finasteride concentration (mg/L)**				
	**Control**	**Solvent control**	**0.003**	**0.03**	**0.3**	**2.5**	**23**
Finasteride	91 (1.1)	89 (0.7)	90 (2.7)	87 (2.8)	87 (1.5)	88 (2.4)	**85 (1.9)**^b^
			**Melengestrol acetate concentration (mg/L)**				
	**Control**	**Solvent control**	**0.005**	**0.05**	**0.3**	**1.9**	**4**
Melengestrol acetate	91 (1.1)	89 (0.7)	86 (0.2)	**82** **(2.1)**^b^	85 (1.2)	**82 (3.3)**^b^	**70 (1.4)**^c^

^a^Concentrations were measured at the beginning and end of each exposure (Table S5)

^b^Although significantly different from pooled controls, this departure represents a 5–8% decrease in survival compared with that of controls, which in light of the 10% acceptable decline in control viability (ASTM 2013), is unlikely to be biologically meaningful

^c^Significant difference from pooled controls

##### Juvenile mussels

Chronic 28-day exposure to FIN significantly reduced the survival of juvenile *L. siliquoidea* at 96 mg/kg (2 mg/L) and both survival and burial ability were reduced at 430 mg/kg (20 mg/L) (Table 3). The aqueous concentrations (e.g., 20 mg/L) presented alongside sediment concentrations are the concentrations measured in the (unspiked) overlying water of sediment exposures. The chronic MGA test was inconclusive due to high mortality in controls (survival was 66% and 72% in the control and solvent control, respectively, which was less than the recommended test acceptability criterion of 80% (ASTM 2013)). Tests were delayed by 2 weeks because the spiked sediments were releasing ammonia at concentrations (≥ 2 mg/L) that could have been toxic to juvenile mussels (the freshwater mussel genus mean acute value for *Lampsilis* is 4.2 mg/L (Augspurger et al. 2003)). Postponement of the tests until ammonia concentrations decreased meant that juvenile mussels were kept in the laboratory for longer than anticipated, which could have compromised the results of the tests. However, during the range-finding test, juvenile mussel survival was 77% after a 21-day exposure to a nominal sediment concentration of 1000 mg/kg MGA, which was significantly lower than the control (Table S2). No chemical analysis was completed during preliminary testing, but we presume that concentrations were similar to those from the subsequent 28-day test, where the measured concentration in the (nominal) 1000 mg/kg treatment was 523 mg/kg (0.6 mg/L).

**Table 3:** Summary of endpoints (mean (standard error of the mean)) for chronic 28-day (juvenile freshwater mussel *Lampsilis siliquoidea*, *n* = 5) or 42-day (amphipod *Hyalella azteca*, *n* = 10, and mayfly *Hexagenia* spp., *n* = 6) exposures to finasteride or melengestrol acetate (mg/kg [dry weight sediment] or mg/L [overlying water], mean measured concentrations^a^). Effect data in bold are significantly different from controls

	**Organism**	**Endpoint**	**Control**	**Solvent control**	**Sediment (mg/kg dw)**					
					**1**	**4**	**11**	**35**	**96**	**430**
					**Water (mg/L)**					
					**0.003**	**0.03**	**0.1**	**0.5**	**2**	**20**
Finasteride	Juvenile mussels	Survival^b^	88 (8.4)	98 (2.0)	nt^c^	94 (2.4)	94 (4.0)	98 (2.0)	**72 (8.6)**^d^	**10 (5.5)**^d^
		Burial ability^e^	77 (11.5)	88 (5.8)	nt	90 (5.5)	98 (2.0)	88 (4.0)	82 (5.5)	**0 (0)**^d^
	Amphipods	Survival^b^	96 (1.3)	96 (1.6)	96 (1.4)	93 (1.7)	94 (1.7)	92 (2.0)	**63 (8.3)**^d^	nt
		Growth^f^	3.4 (0.10)	3.4 (0.09)	3.6 (0.08)	3.6 (0.05)	3.6 (0.10)	3.2 (0.13)	**2.0 (0.19)**^d^	nt
		Young/female	5.6 (0.39)	3.9 (0.55)	4.4 (0.54)	5.8 (0.81)	4.7 (0.83)	4.3 (0.58)	**0.4** **(0.15)**^g^	nt
		Females^h^	53 (3.9)	52 (4.7)	57 (4.2)	53 (3.0)	50 (4.4)	52 (2.7)	**69 (4.4)**^d^	nt
	Mayflies	Survival^b^	98 (1.7)	97 (2.1)	98 (1.7)	97 (2.1)	100 (0.0)	**90 (2.6)**^d,i^	97 (2.1)	nt
		Growth^f^	46 (1.5)	48 (1.7)	45 (0.8)	46 (0.7)	46 (0.9)	48 (1.4)	**40** **(0.8)**^d^	nt
	**Organism**	**Endpoint**	**Control**	**Solvent control**	**Sediment (mg/kg dw)**					
					**1**	**4**	**12**	**37**	**76**	**523**
					**Water (mg/L)**					
					**0.002**	**0.01**	**0.04**	**0.25**	**0.5**	**0.6**
Melengestrol acetate	Juvenile mussels^j^	Survival^b^	66 (8.1)	72 (11.6)	nt	64 (6.8)	67 (7.4)	62 (9.0)	34 (9.8)	57 (6.6)
		Burial ability^e^	66 (14.3)	69 (8.7)	nt	85 (3.9)	83 (5.8)	73 (11.0)	67 (10.7)	88 (8.3)
	Amphipods	Survival^b^	98 (1.8)	96 (1.9)	94 (2.2)	96 (1.8)	95 (1.5)	96 (1.7)	**64 (6.6)**^d^	nt
		Growth^f^	3.3 (0.13)	3.5 (0.15)	3.3 (0.11)	3.7 (0.10)	3.6 (0.08)	3.3 (0.08)	**1.8 (0.12)**^d^	nt
		Young/female	3.6 (0.54)	2.7 (0.45)	2.2 (0.50)	3.8 (0.34)	2.8 (0.40)	2.4 (0.39)	**0.03 (0.025)**^d^	nt
		Females^h^	55 (4.6)	51 (3.3)	52 (3.9)	57 (3.8)	53 (2.5)	50 (3.9)	60 (4.5)	nt
	Mayflies	Survival^b^	90 (6.3)	97 (2.1)	95 (2.2)	102 (1.7)	90 (2.6)	93 (2.1)	92 (4.0)	nt
		Growth^f^	44 (1.6)	43 (0.7)	44 (2.0)	44 (1.0)	43 (1.1)	**38 (0.9)**^d^	**22 (1.1)**^d^	nt

^a^Concentrations were measured at the beginning and end of each exposure (Table S6)

^b^Survival was measured as percent of initial animals added

^c^nt: concentration not tested

^d^Significant difference from pooled controls (*p* < 0.05)

^e^Burial ability was reported as percent of surviving mussels

^f^Growth of amphipods and mayflies was measured as mg wet weight/individual

^g^Significant differences between control and solvent control were observed (30% lower in solvent control); comparisons are based on a significant difference from the solvent control (*p* < 0.05)

^h^The proportion of female amphipods expressed as a percent of surviving amphipods

^i^Although significantly different from pooled controls, this effect was not likely biologically meaningful due to the small magnitude of the response (< 10% lower than pooled controls) and the absence of a concentration-response relationship (no effects observed at the highest concentration)

^j^The test was inconclusive due to mortality greater than the acceptable limit of 80% (ASTM 2013)

##### Gravid mussels

There were no significant changes in the viability of glochidia after a 14-day exposure of gravid female *L. fasciola* to 4.5 mg/kg (0.05 mg/L) FIN or 47 mg/kg (0.2 mg/L) MGA (Fig. S1). No significant chemical-dependent differences in the behaviour of gravid females were observed (Fig. S2). Across treatments, gravid females were observed filtering 55–86% of the time (based on three observations per day), suggesting no sediment or chemical avoidance; however, in several cases, the mussels were partially buried in the sediment, which made some observations difficult. Calculations were adjusted accordingly (i.e., based on the number of instances where observations were possible). Lure displays were observed 20–40% of the time across all treatments.

#### Amphipods

Chronic 42-day exposures to FIN and MGA significantly reduced survival, growth, and reproduction of *H. azteca* at 96 mg/kg (2 mg/L) FIN and 76 mg/kg (0.5 mg/L) MGA. In FIN exposures of 96 mg/kg (2 mg/L), survival, growth, and reproduction of *H. azteca* were 65%, 59%, and 9%, respectively of pooled controls (Table 3). In addition, the proportion of (surviving) adult females exposed to 96 mg/kg (2 mg/L) FIN was significantly higher than that of pooled controls (Table 3). In MGA exposures, survival, growth, and reproduction of *H. azteca* were 66%, 54%, and 0.8%, respectively of those from pooled controls at 76 mg/kg (0.5 mg/L) MGA; however, there were no significant changes in the proportion of adult females (Table 3).

#### Mayflies

In 42-day exposures, mayfly survival was significantly lower than pooled controls at 35 mg/kg (0.5 mg/L) FIN; however, given the small magnitude of the difference (< 10%) and the absence of a concentration-response relationship (no significant effect on survival at 96 mg/kg [2 mg/L]), this is unlikely to be a biologically relevant effect (Table 3). FIN significantly reduced mayfly growth at the highest test concentration; mayflies exposed to 96 mg/kg (2 mg/L) FIN were 14% smaller than the pooled controls (Table 3). Exposure to MGA did not affect survival; however, mayflies exposed to 37 mg/kg (0.25 mg/L) and 76 mg/kg (0.5 mg/L) MGA were significantly smaller, 12 and 49% of the pooled controls, respectively (Table 3).

### Modelling

#### Fugacity

In a scenario of homogeneous discharge (equal discharge to air, water, and soil), the level III multimedia fugacity models predicted the majority of these compounds to be retained in soils (89% of FIN and 79% of MGA, respectively), and between 5 and 11% of both FIN and MGA to be retained in water and sediment (Table S3). In a scenario of 100% discharge to water, the level III multimedia fugacity models predicted approximately equal proportions (between 46 and 54%) of each contaminant in water and sediment (Table S3). Lastly, in a scenario of 100% discharge to soil, the models predicted more than 99% of the chemicals to be retained in soil, with about 0.1% of the compounds being detected in water and sediment (Table S3). In all cases, the proportion of compound distributed to the air compartment was negligible.

#### Toxicity estimation

The ECOSAR model classified FIN into the acrylamide and the amide functional groups for the class-based quantitative structure-activity relationships (QSARs), with the acrylamide yielding the most conservative (e.g., more toxic) estimates. Predicted acute toxicity values (48–96 h LC50) for fish, daphnids, and mysids varied between 1.1 and 6.09 mg/L (Table S4). Predicted ChVs varied between 0.0007 and 0.1 mg/L (Table S4). MGA was classified into the esters, vinyl/allyl/propargyl ketones, and vinyl/allyl/propargyl ester QSAR classes. The vinyl/allyl/propargyl ester class yielded more conservative estimates of toxicity, with acute toxicity values (48–96 h LC50) for fish and daphnids of 0.1 and 3.0 mg/L, respectively (Table S4). The ChVs for fish and daphnids were 0.007 and 0.01 mg/L, respectively (data not available for mysids; Table S4).

### Chemical analysis

Measured water concentrations in the glochidia aqueous exposures were generally similar (i.e., 90–124% of nominal) to the nominal concentrations during the FIN experiment. For the MGA experiment, more pronounced differences were observed (i.e., 17–190% of nominal), because measured concentrations were greater than nominal concentrations in the two lowest treatments and were lower than nominal at the highest concentration, where the measured concentration was 4 mg/L rather than 25 mg/L (Table S5). We suspect that MGA reached the water solubility limit (estimated at 0.54 to 1.89 mg/L (US EPA 2013); hence the use of a solvent carrier; Table 1), as we noticed precipitation at the bottom of the vessels in the 25 mg/L (nominal) treatment only. Measured sediment concentrations in the exposures with juvenile mussels, amphipods, and mayflies were 31–43% and 25–52% of nominal concentrations for FIN and MGA, respectively (Table S6). Overlying water concentrations in FIN sediment exposures were 0.003–20 mg/L and increased with sediment concentration (Table S6). While overlying water concentrations of MGA were similar to those of FIN at the lowest concentrations (0.002–0.25 mg/L), they levelled off at the highest two concentrations (0.5–0.6 mg/L), likely due to solubility limits (Table S6). Sediment concentrations in the gravid female mussel experiment were 42 ± 8.5 and 47 ± 4.2% of nominal concentrations for FIN and MGA, respectively (Table S5), and overlying water concentrations (0.05 and 0.2 mg/L, respectively) were comparable with those from sediment tests with juvenile mussels, amphipods, and mayflies (Tables S5, S6).

## Discussion

In the present study, the toxicity of two endocrinologically active pharmaceuticals was assessed using a variety of model organisms, including benthic, epibenthic, and filter-feeding invertebrates. Finasteride and MGA were not particularly toxic to the test species used in this study. The ChV for FIN was 7.6 mg/L for glochidia and 58 mg/kg (1 mg/L) for juvenile freshwater mussels, amphipods, and mayflies (Table 4); for mayflies, growth was more sensitive than survival (Table 4). Melengestrol acetate was slightly more toxic than FIN, with a ChV of 2.8 mg/L for glochidia, 53 mg/kg (0.4 mg/L) for amphipods, and 21 mg/kg (0.1 mg/L) for mayflies (Table 4). Juvenile freshwater mussels appeared less sensitive than amphipods and mayflies (Table 4). The juvenile mussel sediment test with MGA was inconclusive due to high control mortality, though a range-finding test indicated that effects were not observed at concentrations below 1000 mg/kg (nominal; Table S2), and the sub-chronic exposure to 47 mg/kg (0.2 mg/L) MGA had no significant effects on the behaviour or filtering activity of gravid *L. fasciola* or their brooding glochidia (Figs. S1 and S2). Mayflies were the most sensitive species to MGA in the sediment exposures, with a significant reduction in growth observed at 37 mg/kg (0.25 mg/L) MGA (Table 3). Differences in sensitivity between these species have been reported for other compounds; although glochidia are considerably more sensitive than *H. azteca* to some inorganic contaminants such as copper, ammonia, and chloride (Wang et al. 2007; Bartelt-Hunt et al. 2012; Gillis 2011), they have been reported to be less sensitive than amphipods and mayflies to organic contaminants (e.g., substituted phenylamine antioxidants (Prosser et al. 2017b), neonicotinoid insecticides (Bartlett et al. 2018, 2019; Prosser et al. 2016; Salerno et al. 2018)).

**Table 4:** No-observed effect concentrations (NOEC), lowest-observed effect concentrations, and chronic values (ChV, geometric mean of the NOEC and LOEC) for acute (48 h freshwater mussel *Lampsilis siliquoidea* glochidia, *n* = 4) and chronic 28-day (juvenile freshwater mussel *Lampsilis siliquoidea*, *n* = 5) or 42-day (amphipod *Hyalella azteca*, *n* = 10, and mayfly *Hexagenia* spp., *n* = 6) exposures to finasteride or melengestrol acetate (mg/kg [dry weight sediment] or mg/L [overlying water], measured concentrations)

		Finasteride			Melengestrol acetate		
Organism	Most sensitive endpoint	NOEC	LOEC	ChV	NOEC	LOEC	ChV
Freshwater mussel glochidia	Viability	2.5 mg/L	23 mg/L^a^	7.6 mg/L	1.9 mg/L^b^	4 mg/L^b^	2.8 mg/L
Freshwater mussel juveniles	Survival	35 mg/kg/0.5 mg/L	96 mg/kg/2 mg/L	58 mg/kg/1 mg/L	NC^c^	NC	NC
Amphipods	Survival, growth^d^	35 mg/kg/0.5 mg/L	96 mg/kg/2 mg/L	58 mg/kg/1 mg/L	37 mg/kg/0.25 mg/L	76 mg/kg/0.5 mg/L	53 mg/kg/0.3 mg/L
Mayflies	Growth	35 mg/kg/0.5 mg/L	96 mg/kg/2 mg/L	58 mg/kg/1 mg/L	12 mg/kg/0.04 mg/L	37 mg/kg/0.25 mg/L	21 mg/kg/0.1 mg/L

^a^Although a statistically significant difference was observed at 23 mg/L, glochidia viability was decreased by 5% compared with the controls (90%)

^b^Although statistically significant, glochidia viability was decreased by 5–8% at concentrations of 0.05 and 1.9 mg/L, which is within the range permitted for controls and unlikely to be biologically significant. A more pronounced decrease in glochidia viability was observed at a concentration of 4 mg/L and was more appropriate to use as the LOEC

^c^NC: not calculated, test inconclusive due to mortality greater than the acceptable limit of 80% (ASTM 2013)

^d^Survival, growth, number of young per female, and number of female (finasteride exposure only) were all significantly different from controls at the same concentration; however, reproduction and sexual maturation are dependent on growth, and it was not possible to determine if the number of young/female and number of females were due to the presence of smaller, immature animals in the highest treatment. Therefore, only survival and growth endpoints were specified for the NOECs and LOECs

Although there are far fewer studies on the sensitivities of freshwater mussels to PPCPs than to inorganic contaminants, the limited studies indicate that for some PPCPs, mussels are more sensitive than other aquatic organisms, while for other PPCPs, the sensitivity is comparable. Gilroy et al. (2017) reported LC50s of 0.3 and 0.4 mg/L for the SSRI amitriptyline in a 24-h test with glochidia and a 14-day test with juvenile *L. siliquoidea*, respectively, as well as a 24-h LC50 of 0.06 mg/L for glochidia and a 28-day LC50 of 0.04 mg/L for juvenile *L. siliquoidea* for sertraline. In comparison, Minguez et al. (2014) reported 48-h *D. magna* EC50s of 4.8 mg/L for amitriptyline, and 1.2 mg/L for sertraline, suggesting a difference in sensitivity of at least an order of magnitude.

In the present study, sublethal endpoints in amphipods were affected by both FIN and MGA. Reduced growth and reproduction were detected at the highest test concentrations of FIN and MGA. There is a strong relationship between growth and reproduction in *H. azteca*; reduced growth is likely to result in decreased reproduction (larger individuals have higher reproductive output) and delayed sexual maturity (reproduction requires a minimum body size) (Ingersoll et al. 1998; Moore and Farrar 1996; Soucek et al. 2016). As growth and reproduction were both significantly lower at the highest test concentrations of FIN and MGA, we were unable to determine if these resulted from direct effects on reproduction, or direct effects on growth which indirectly affected reproduction. The proportion of female *H. azteca* (as determined by the absence of male secondary sex characteristics) increased significantly following chronic FIN exposure to the highest concentration tested (96 mg/kg (2 mg/L)), whereas no effects on the proportion of females was observed following exposure to MGA (up to 76 mg/kg or 0.5 mg/L; Table 3). Altered sex ratios can arise upon exposure to estrogenic or antiandrogenic compounds from either sex-specific mortality (e.g., Versonnen et al. 2004) or altered sexual differentiation (e.g., Lor et al. 2015). It is unclear whether the observed change in sex ratio was due to sex-biased mortality, alteration of secondary sexual characteristics, or reduced growth/delayed development. An additional complication is that growth was reduced following exposure to both FIN and MGA (Table 3), and it is difficult to differentiate males from females if males are small and secondary sex characteristics are not fully developed.

There is little information available on the fate and behaviour of either FIN or MGA in aquatic environments. The level III multimedia fugacity models confirmed the capacity of soils to retain both compounds, as both the equal discharges and soil discharges models predicted 79–99% of the compounds would be retained in soils. In a water-only discharge scenario, sediments could be a repository for approximately half of FIN and MGA released. In the present study, FIN and MGA were spiked into sediments but were measured in the overlying water as well as the sediment, indicating that desorption occurred. The mass of compound dissolved in the overlying water usually represented less than 10%, and at most 14% (1000 mg/kg FIN) of the spiked compound. Given their log K_ow_ values (3.2–4.4), these compounds may also have been bioaccumulated and bioconcentrated by the benthic organisms, although this was beyond the scope of the present study. In addition, chemical analysis focused on FIN and MGA, and did not include potential metabolites. Both FIN and MGA are actively metabolized in humans and bovines, respectively (Cooper et al. 1967; Lemke and Williams 2008). Furthermore, MGA readily undergoes direct photolysis under both natural and simulated sunlight, with half-lives of approximately 45 min (Qu et al. 2012); FIN does not appear to be subject to photolysis, and its half-life in river water appears to vary between 11 and > 28 days (Blum et al. 2017). Thus, in the present study with spiked sediments, FIN and MGA may also have been metabolized or degraded via biotic or abiotic processes.

Given the paucity of data on the effects of FIN and MGA on non-target species, the toxicity of each compound to aquatic organisms was estimated using structure-activity relationships. The conservative estimates obtained through ECOSAR for acute toxicity (i.e., LC50s/EC50s) to fish, daphnids, and mysids (1.11–6.09 mg/L for FIN, 0.1–3 mg/L for MGA; Table S4) are lower than the water-only toxicity to glochidia in the present study, where we observed a decrease in viability of 20% at 4 mg/L for MGA and no biologically meaningful differences at concentrations ≤ 23 mg/L for FIN. The existing data on acute toxicity of FIN and MGA indicate toxicity (if any) is observed in the mg/L range, agreeing with the results of the present study, although this interpretation should be made with caution as we were unable to calculate LC50s from our data. The 96-h LC50s of FIN for rainbow trout (*Oncorhynchus mykiss*) and *Daphnia magna* were 20 and 21 mg/L, respectively (FDA-CDER 1996). *Daphnia magna* was also fairly insensitive to MGA, with no significant difference in mobility reported after a 48-h exposure to 2 mg/L. Chronic toxicity data for both compounds are more scarce. To our knowledge, there is no information available in the scientific literature on the chronic toxicity of FIN to aquatic species, and only two studies have been published for MGA. No toxicity was observed in goldfish (*Carassius auratus*) exposed to 1 mg/L MGA for 21 days (Upjohn Company 1996). In the African clawed frog (*Xenopus laevis*), larvae were significantly smaller (length and weight) than controls after a chronic exposure to 100 ng/L MGA, although no mortality or developmental effects were observed (Finch et al. 2013). Organisms in the chronic tests from the present study were exposed to chemicals present in both the overlying water and sorbed to sediments, making comparisons with QSARs (exposures via water only) difficult. Nevertheless, we calculated FIN ChVs of 57 mg/kg dw (1 mg/L) for juvenile mussels, amphipods, and mayflies, respectively, and MGA ChVs of 53 mg/kg dw (0.4 mg/L) and 21 mg/kg dw (0.1 mg/L) for amphipods and mayflies, respectively. In comparison, the modelled ChVs of 0.0007–0.1 mg/L are conservative.

Freshwater molluscs are amongst the most imperilled group of aquatic organisms due to contributing factors such as habitat alteration, invasive species, and poor water quality (Lydeard et al. 2004). Due to their unique life history, the exposure of unionid mussels to contaminants varies among different life stages. As glochidia, mussels are directly exposed to surface water prior to their attachment to a vertebrate host, whereas juvenile and adult stages are exposed to both sediment and surface water when they burrow in the sediments and filter-feed. In the present study, we assessed the toxicity of FIN and MGA at three life stages of the freshwater mussels in the medium most relevant to each life stage; glochidia were tested in aqueous exposures, whereas juvenile and adult gravid female mussels were tested in sediment exposures. Effects, if any, were observed at concentrations orders of magnitude greater (e.g., mg/L) than those expected in aquatic environments (e.g., ng/L to low μg/L). Both FIN and MGA did not appear to affect the behaviour or the brooding glochidia of gravid females (Figs. S1 and S2).

Previous studies have found that PPCPs can alter the behaviour of exposed adult freshwater mussels. Leonard et al. (2014, 2017) assessed the effects of the synthetic hormone 17α-ethinylestradiol (EE2) on adult *L. fasciola* and *Elliptio complanata*, and reported changes in the frequency of foot protrusion (males), siphoning activity, and release of a greater proportion of immature eggs (females). Studies on the effects of the selective serotonin reuptake inhibitor fluoxetine also reported induction of spawning behaviour (Bringolf et al. 2010; Hazelton et al. 2013) and increased lure display and locomotory behaviour, albeit at concentrations 10–100 times higher than those expected in the environment (Hazelton et al. 2014).

In other invertebrates, studies with antiandrogenic drugs reported effects at concentrations in the mg/L range. For example, concentrations of flutamide up to 1 mg/L delayed the maturation of female *D. magna* and inhibited the embryonic development of neonates, resulting in abortions; the 48-h EC50 for immobilization was 2.7 mg/L (Haeba et al. 2008). Exposure of the freshwater snail *Marisa cornuarietis* to cyproterone acetate at 1.25 mg/L reduced the size of male sex organs in juveniles, although this effect was reversible after puberty (Tillmann et al. 2001). Further, Tillmann et al. (2001) reported that the antiandrogenic response of cyproterone acetate was much reduced compared with the magnitude of responses to synthetic estrogens (ethinylestradiol) or androgens (methyltestosterone).

The results of the present study are in agreement with the limited toxicity data on these compounds in the literature and the predicted toxicity from modelling simulations, all of which indicate that FIN and MGA do not appear to pose a direct risk to aquatic invertebrates at concentrations expected in the environment. However, laboratory tests using individual compounds may not accurately reflect the complex mixtures of chemicals to which aquatic organisms are exposed in the environment, and further research on environmentally relevant mixtures of PPCPs will be important in determining the risk to aquatic invertebrate populations.

## Conclusion

In the present study, we assessed the toxicity of FIN and MGA in acute, aqueous exposures with mussel larvae, and chronic sediment exposures with mussels (juvenile and adult), amphipods, and mayflies. Toxicity of FIN and MGA in these test species, if any, occurred at 96 and 37 mg/kg or higher in sediment, and 2 and 0.25 mg/L or higher in water, respectively. Given this low toxicity in comparison with concentrations expected in aquatic environments (range of ng/L to μg/L), effects on survival, growth, reproduction, and behaviour are not expected in natural populations of these species exposed to FIN or MGA.

## Electronic supplementary material

ESM 1 (DOCX 538 kb)
