# Supplementary material for: Toxicity of the pharmaceuticals finasteride and melengestrol acetate to benthic invertebrates
Source: Environ Sci Pollut Res Int. 2020 Jul 22;27(33):41803–15. doi: 10.1007/s11356-020-10121-7 (PMC7679302; doi:10.1007/s11356-020-10121-7)
Supplement: Supplementary file 1 — (DOCX 538 kb) [file 11356_2020_10121_MOESM1_ESM.docx]

Supplemental Information

# Toxicity of the Pharmaceuticals Finasteride and Melengestrol Acetate to Benthic Invertebrates

Ève AM Gilroy^1,2*^, Adrienne J Bartlett^2^, Patricia L Gillis^2^, Amanda M Hedges^2^, Lisa R Brown^2^, Nicholas A Bendo^3^, Joseph Salerno^2^, Emily AM Holman^2^, Naomi L Stock^4^, and Shane R de Solla^3^

^1^ Green House Science, Burlington, ON
^2^ Aquatic Contaminant Research Division, Environment and Climate Change Canada, Burlington, ON
^3^ Ecotoxicology and Wildlife Health Division, Environment and Climate Change Canada, Burlington, ON

^4^ Water Quality Centre, Trent University, Peterborough, ON

* Current address: Aquatic Contaminant Research Division, Environment and Climate Change Canada, Burlington, ON, L7S 1A1. Phone: 905-336-4603, Email: eve.gilroy@canada.ca

**Table S1** Water quality parameters collected during the toxicity studies with the freshwater mussels *Lampsilis siliquoidea (*glochidia and juvenile mussels) and *Lampsilis fasciola* (gravid females), the amphipod *Hyalella azteca* and the mayfly *Hexagenia spp*.

| Species | Test | n | pH | Temperature  (°C) | Dissolved  Oxygen (mg/L) | Conductivity  (µS/cm) | Chloride  (mg/L) | Ammonia  (mg/L) |
| --- | --- | --- | --- | --- | --- | --- | --- | --- |
| Glochidia | 48 h | 15 | 8.3 ± 0.03 | NM | 8.2 ± 0.35 | 346 ± 40 | NM | ND |
| Juvenile mussels | 21 d | 21 | 8.4 ± 0.11 | 20 ± 1.2 | 8.4 ± 0.48 | 450 ± 94 | NM | ND |
| Juvenile mussels | 28 d | 72 | 8.1 ± 0.10 | 20 ± 0.5 | 8.9 ± 0.21 | 485 ± 79 | 29 ± 9.1 | 0.2 ± 0.29 |
| Gravid mussels | 14 d | 25 | 7.9 ± 0.11 | 18 ± 0.8 | 9.1 ± 0.29 | 471 ± 52 | 42 ± 6.1 | 1.3 ± 1.01 |
| Amphipods | 21 d | 84 | 8.5 ± 0.05 | NM^a^ | 8.3 ± 0.48 | 490 ± 4 | NM | ≤ 0.5 |
| Amphipods | 42 d | 336 | 8.5 ± 0.12 | NM^a^ | 8.4 ± 0.68 | 510 ± 4 | NM | ≤ 1.0 |
| Mayflies | 21 d | 84 | 8.5 ± 0.06 | NM^a^ | 8.0 ± 0.41 | 480 ± 4 | NM | ≤ 0.5 |
| Mayflies | 42 d | 168 | 8.5 ± 0.11 | NM^a^ | 8.3 ± 0.28 | 520 ± 7 | NM | ≤ 0.5 |

NM = not measured

ND = not detected

^a^ Incubators were maintained at 23-25 °C

# Range-finding tests

Range-finding tests of 21 d were completed with juvenile mussels, amphipods and mayflies to assist with the selection of the concentrations to be used in final testing. Nominal concentrations in range-finding tests were 0.1, 1, 10, 100, and 1000 mg/kg, based on dry weight of sediment. Range-finding test conditions were identical to those described in the manuscript for chronic sediment tests with the exception of test duration.

## Juvenile mussels

Ten juvenile mussels were added to each beaker (three replicates per concentration), and fed 200 µL of an algae mixture (4.5·10^8^ cells Nanno 3600 - *Nannochloropsis*) and 0.5·10^8^ cells Shellfish Diet 1800 (Reed Mariculture Inc., Campbell, CA) twice daily on weekdays, and daily on weekends. After 21 d, mussels were recovered and transferred to Petri dishes filled with clean sand and housed in large trays of aerated culture water to assess burial ability. Mussel behaviour was observed daily for 72 h, and those that were widely gaping or showed no foot movement were considered dead.

## Amphipods

Fifteen juvenile amphipods (3-11 days old) were added to each beaker (three replicates for each control and PPCP concentration). Amphipods were fed 2.5 mg Tetra-min twice per week during weeks one and two, and three times during week three. At the end of the test, amphipods were removed and the survivors were counted and weighed.

## Mayflies

Ten mayflies (5-8 mg wet weight) were added to each beaker (three replicates for each control and PPCP concentration). Mayflies were fed 50 mg of a mixture of cereal wheat grass, Brewer’s yeast, and ground Tetra-min per beaker per week (Bartlett et al. 2018). At the end of the test, survival and wet weight of mayflies in each replicate were recorded.

**Table S2** Survival (%) and burial (%) or growth (mg wet weight; mean (standard error of the mean), *n*= 3) of juvenile freshwater mussels *Lampsilis siliquoidea*, amphipods *Hyalella azteca* and mayflies *Hexagenia spp.* after a 21 d range-finding test to finasteride or melengestrol acetate (mg/kg dry weight, nominal concentrations).

|  | Organism | Endpoint | Control | Solvent Control | 0.1 | 1 | 10 | 100 | 1000 |
| --- | --- | --- | --- | --- | --- | --- | --- | --- | --- |
| Finasteride | Juvenile mussels  *L. siliquoidea* | Survival | 100 (0.0) | 97 (3.3) | 100 (0.0) | 100 (0.0) | 100 (0.0) | 100 (0.0) | 53 (6.7)^a^ |
|  |  | Burial | 83 (12.0) | 66 (8.3) | 72 (6.3) | 93 (3.3) | 87 (6.7) | 90 (0.0) | 77 (5.1) |
|  | Amphipods  *H. azteca* | Survival | 96 (4.4) | 100 (0.0) | 100 (0.0) | 100 (0.0) | 100 (0.0) | 100 (0.0) | 0^a^ (0.0) |
|  |  | Growth | 0.9^b^ (0.07) | 1.1 (0.01) | 1.1 (0.12) | 1.1 (0.04) | 1.2 (0.05) | 0.79 (0.06) | n/a^c^ |
|  | Mayflies  *Hexagenia* spp. | Survival | 100 (0) | 100 (0.0) | 100 (0.0) | 100 (0.0) | 100 (0.0) | 93 (3.3)^a^ | 3.3^a^ (3.3) |
|  |  | Growth | 33 (1.3) | 31 (0.5) | 30 (0.5) | 30 (0.6) | 31 (0.3) | 29 (1.3) | 4.5^a,d^ (0.0) |
| Melengestrol Acetate | Juvenile mussels  *L. siliquoidea* | Survival | 97 (3.3) | 87 (3.3) | 93 (3.3) | 97 (3.3) | 97 (3.3) | 100 (0.0) | 77 (8.8) |
|  |  | Burial | 88 (7.3) | 69 (9.3) | 85 (4.8) | 97 (7.0) | 90(5.8) | 90 (0.0) | 68 (5.8) |
|  | Amphipods  *H. azteca* | Survival | 98 (2.2) | 95 (5.9) | 100 (3.8) | 100 (0.0) | 95 (2.2) | 95 (2.2) | 0^a^ (0.0) |
|  |  | Growth | 1.0 (0.12) | 1.0 (0.26) | 1.0 (0.09) | 1.0 (0.07) | 1.1 (0.10) | 0.7 (0.15) | n/a^c^ |
|  | Mayflies  *Hexagenia* spp. | Survival | 90 (5.8) | 97 (3.3) | 100 (0.0) | 97 (3.3) | 100 (0.0) | 100 (0.0) | 37^a^ (8.8) |
|  |  | Growth | 32 (0.3) | 30 (0.7) | 33 (0.2) | 30 (0.9) | 31 (1.2) | 20^a^ (0.7) | 7.4^a^ (0.24) |

^a^ Significant difference from pooled controls (*p* < 0.05).

^b^ Significant difference from solvent control (*p* < 0.05).

^c^ No surviving animals, therefore growth could not be determined.

^d^ Only one replicate with surviving animals.

**Table S3** Modelling results of Level III multimedia fugacity models using US EPA’s Episuite (US EPA 2013), for finasteride and melengestrol acetate, based on discharges equally divided between air, soil, and water, discharges solely to water, and discharges solely to soil.

|  | Finasteride | Melengestrol Acetate |
| --- | --- | --- |
| Discharge to Air, Water, Soil | | |
| Air | 0.02% | - |
| Water | 4.95% | 11% |
| Soil | 89.2% | 79.3% |
| Sediment | 5.86% | 9.73% |
| 100% Discharge to Water | | |
| Air | - | - |
| Water | 45.8 % | 53% |
| Soil | - | - |
| Sediment | 54.2% | 47% |
| 100% Discharge to Soil | | |
| Air | - | - |
| Water | 0.09% | 0.06% |
| Soil | 99.8% | 99.9% |
| Sediment | 0.11% | 0.06% |

**Table S4** ECOSAR simulation results for the toxicity of finasteride and melengestrol acetate to fish, daphnids and mysids. SW = salt water species, ChV = chronic value, N/A = data not available.

| Chemical | Organism | Endpoint | Value  (mg/L) | Chemical Class retained |
| --- | --- | --- | --- | --- |
| Finasteride | Fish | 96 h LC50 | 2.17^a^ | Acrylamides |
|  | Daphnid | 48 h LC50 | 6.09^a^ |  |
|  | Mysid (SW) | 96 h LC50 | 1.11 |  |
|  | Fish | ChV | 0.10 |  |
|  | Daphnid | ChV | 0.07 |  |
|  | Mysid (SW) | ChV | 0.0007 |  |
| Melengestrol Acetate | Fish | 96 h LC50 | 0.1 | Vinyl/allyl/propargyl Esters^b^ |
|  | Daphnid | 48 h LC50 | 3.0^a^ |  |
|  | Mysid (SW) | N/A | |  |
|  | Fish | ChV | 0.007 |  |
|  | Daphnid | ChV | 0.01 |  |
|  | Mysid (SW) | N/A | |  |

^a^ Chemical may not be soluble enough to measure this predicted effect. If the effect level exceeds the water solubility by 10X, typically no effects at saturation (NES) are reported.

^b^ Chemical class for which data were most conservative, and hence retained

**Table S5** Water concentrations (mean ± standard deviation (*n*)) of finasteride and melengestrol acetate during testing with glochidia from the freshwater mussel *Lampsilis siliquoidea*.

|  | Nominal concentration  (mg/L) | Measured concentration  (mg/L) | % of nominal |
| --- | --- | --- | --- |
| Finasteride | Control | < MLOQ (2)^a^ | - |
|  | Solvent Control | < MLOQ (2) | - |
|  | 0.0025 | 0.003 ± 0.0012 (2) | 118 |
|  | 0.025 | 0.03 ± 0.005 (2) | 122 |
|  | 0.25 | 0.3 ± 0.04 (2) | 124 |
|  | 2.5 | 2.5 ± 0.00 (2) | 100 |
|  | 25 | 23 ± 2.1 (2) | 90 |
| Melengestrol Acetate | Control | < MLOQ (5) | - |
|  | Solvent Control | < MLOQ (2) | - |
|  | 0.0025 | 0.005 ± 0.0006 (2) | 190 |
|  | 0.025 | 0.05 ± 0.002 (2) | 182 |
|  | 0.25 | 0.3 ± 0.06 (2) | 120 |
|  | 2.5 | 2 ± 0.4 (2) | 74 |
|  | 25 | 4 ± 3.6 (2)^b^ | 17 |

^a^ Method limit of quantitation

^b^ Precipitation was observed at the bottom of the vessels, suggesting the limit of solubility had been exceeded.

**Table S6** Water and sediment concentrations of finasteride and melengestrol acetate (mean ± standard deviation (*n*)) at the beginning (prior to addition of organisms) and end of exposures with juvenile freshwater mussel *Lampsilis siliquoidea* (28 d), amphipod *Hyalella azteca* (42 d) and mayfly *Hexagenia* spp. (42 d). The data for all experiments were combined to produce average concentrations.

|  | Nominal sediment concentration  (mg/kg dw) | Measured water concentration  (mg/L) |  | Measured sediment concentration  (mg/kg dw) |  | % of nominal |
| --- | --- | --- | --- | --- | --- | --- |
|  |  | Beginning | End | Beginning | End |  |
| Finasteride | Control | < MLOQ (2)^a^ | < MLOQ (3) | < MLOQ (2)^a^ | < MLOQ (3) | - |
|  | Solvent Control | < MLOQ (2) | < MLOQ (3) | < MLOQ (2) | < MLOQ (3) | - |
|  | 3 | 0.006 (1) | 0.002 ± 0.0006 (2) | 1.2 (1) | 0.8 ± 0.01 (2) | 27-40 |
|  | 10 | 0.03 ± 0.008 (2) | 0.03 ± 0.013 (3) | 4 ± 0.4 (2) | 3.8 ± 0.31 (3) | 38-41 |
|  | 30 | 0.1 ± 0.04 (2) | 0.1 ± 0.10 (3) | 13 ± 1.4 (2) | 10 ± 1.53 (3) | 34-43 |
|  | 100 | 0.6 ± 0.15 (2) | 0.5 ± 0.21 (3) | 36 ± 0.0 (2) | 34 ± 5.6 (3) | 34-36 |
|  | 300 | 2.6 ± 1.41 (2) | 2 ± 1.48 (3) | 103 ± 16.3 (2) | 92 ± 9.5 (3) | 31-34 |
|  | 1000 | 16 (1) | 23 (1) | 488 (1) | 371 (1) | 37-49 |
| Melengestrol Acetate | Control | < MLOQ (3)^b^ | < MLOQ (2) | < MLOQ (3) | < MLOQ (2) | - |
|  | Solvent Control | < MLOQ (3) | < MLOQ (2) | < MLOQ (3) | < MLOQ (2) | - |
|  | 3 | 0.004 (1) | 0.001 ± 0.0003 (2) | 1.8 (1) | 0.9 ± 0.48 (2) | 29-60 |
|  | 10 | 0.01 ± 0.005 (3) | 0.002 ± 0.0023 (2) | 4.6 ± 0.79 (3) | 2.5 ± 0.92 (2) | 25-46 |
|  | 30 | 0.06 ± 0.019 (3) | 0.01 ± 0.011 (2) | 15 ± 3.0 (3) | 9 ± 2.8 (2) | 30-49 |
|  | 100 | 0.37 ± 0.045 (3) | 0.14 ± 0.072 (2) | 35 ± 6.5 (3) | 31 ± 7.1 (2) | 31-35 |
|  | 300 | 0.5 ± 0.10 (3) | 0.5 ± 0.04 (2) | 82 ± 6 (3) | 67 ± 3 (3) | 22-27 |
|  | 1000 | 0.6 ± 0.06 (2) | No sample | 523 ± 1 (2) | No sample | 52 |

^a^ Method limit of quantitation

^b^ One sample had a concentration of melengestrol acetate (0.0003 mg/L) above method limit of quantitation (MLOQ = 0.0002 mg/L)

**Table S7** Water concentrations of finasteride or melengestrol acetate (mean ± standard deviation (*n*)) at the beginning (prior to addition of organisms) and end of testing (14 d) with gravid freshwater mussels *Lampsilis fasciola*.

|  | Nominal sediment concentration  (mg/kg dw) | Measured water concentration  (mg/L) | Measured sediment concentration  (mg/kg dw) | % of nominal |
| --- | --- | --- | --- | --- |
| Finasteride | Control | < MLOQ (2)^a^ | < MLOQ (2) | - |
|  | Solvent Control | < MLOQ (2) | < MLOQ (2) | - |
|  | 10 | 0.05 ± 0.008 (2) | 4.2 ± 0.85 (2) | 42 |
| Melengestrol Acetate | Control | < MLOQ (2) | < MLOQ (2) | - |
|  | Solvent Control | < MLOQ (2) | < MLOQ (2) | - |
|  | 100 | 0.2 ± 0.05 (2) | 47 ± 4.2 (2) | 47 |

^a^ Method limit of quantitation


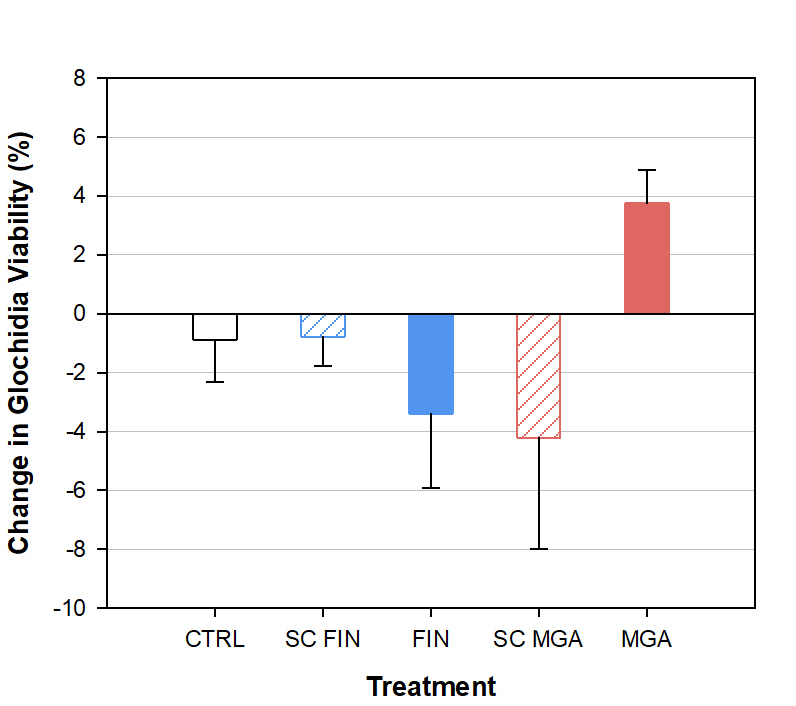


**Figure S1** Variation of glochidia viability in gravid mussels (*Lampsilis fasciola*) (mean ± standard error of the mean, *n* = 5) during a 14 d sediment exposure to 10 mg/kg dw finasteride (FIN) or 100 mg/kg dw melengestrol acetate (MGA). CTRL = negative control, SC = solvent control. No differences between treatments were observed (*p* = 0.051, Kruskal-Wallis ANOVA on ranks). It should also be noted that variations were of less than 10%, the tolerated change in glochidia viability for test acceptability (ASTM 2013).


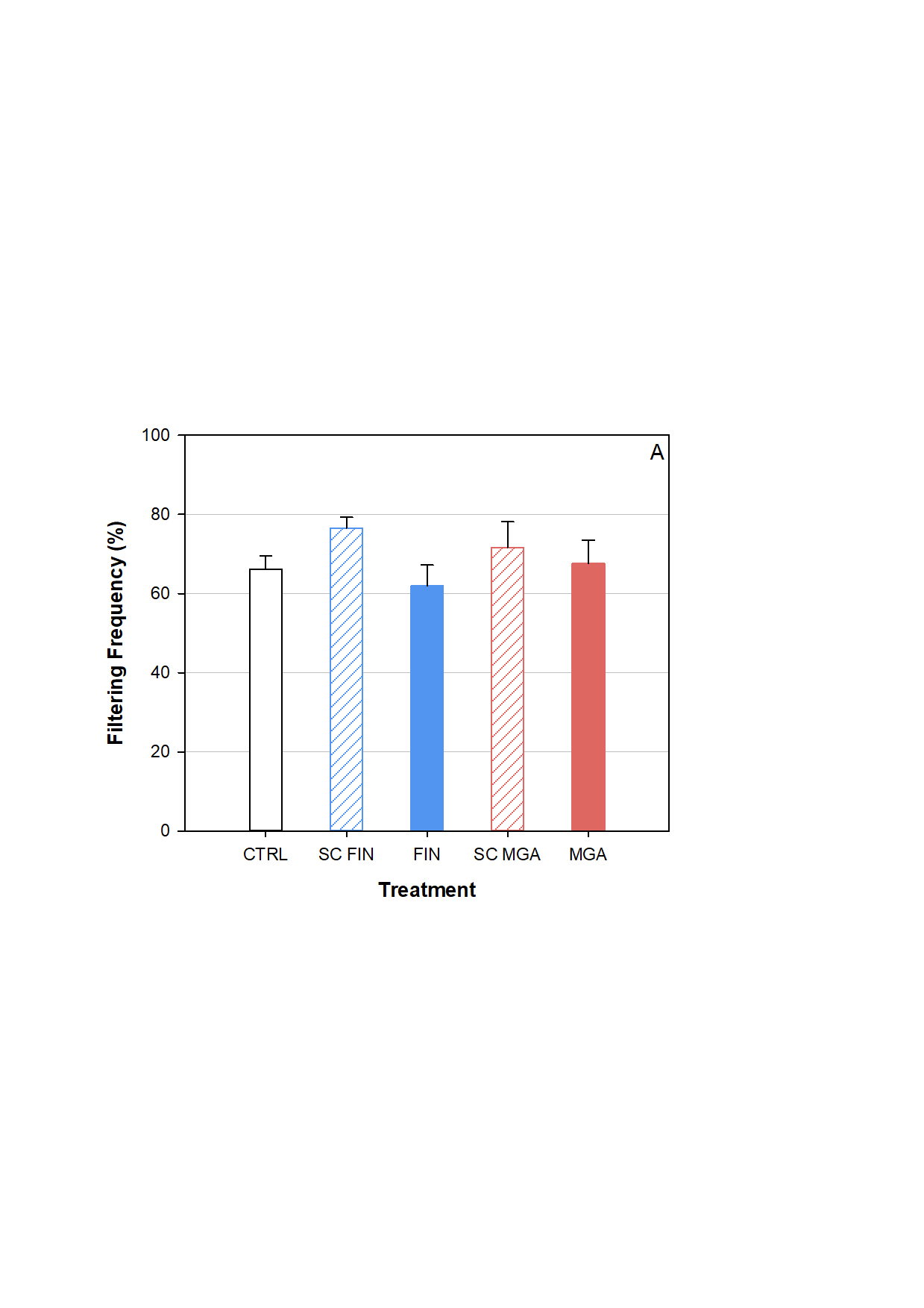


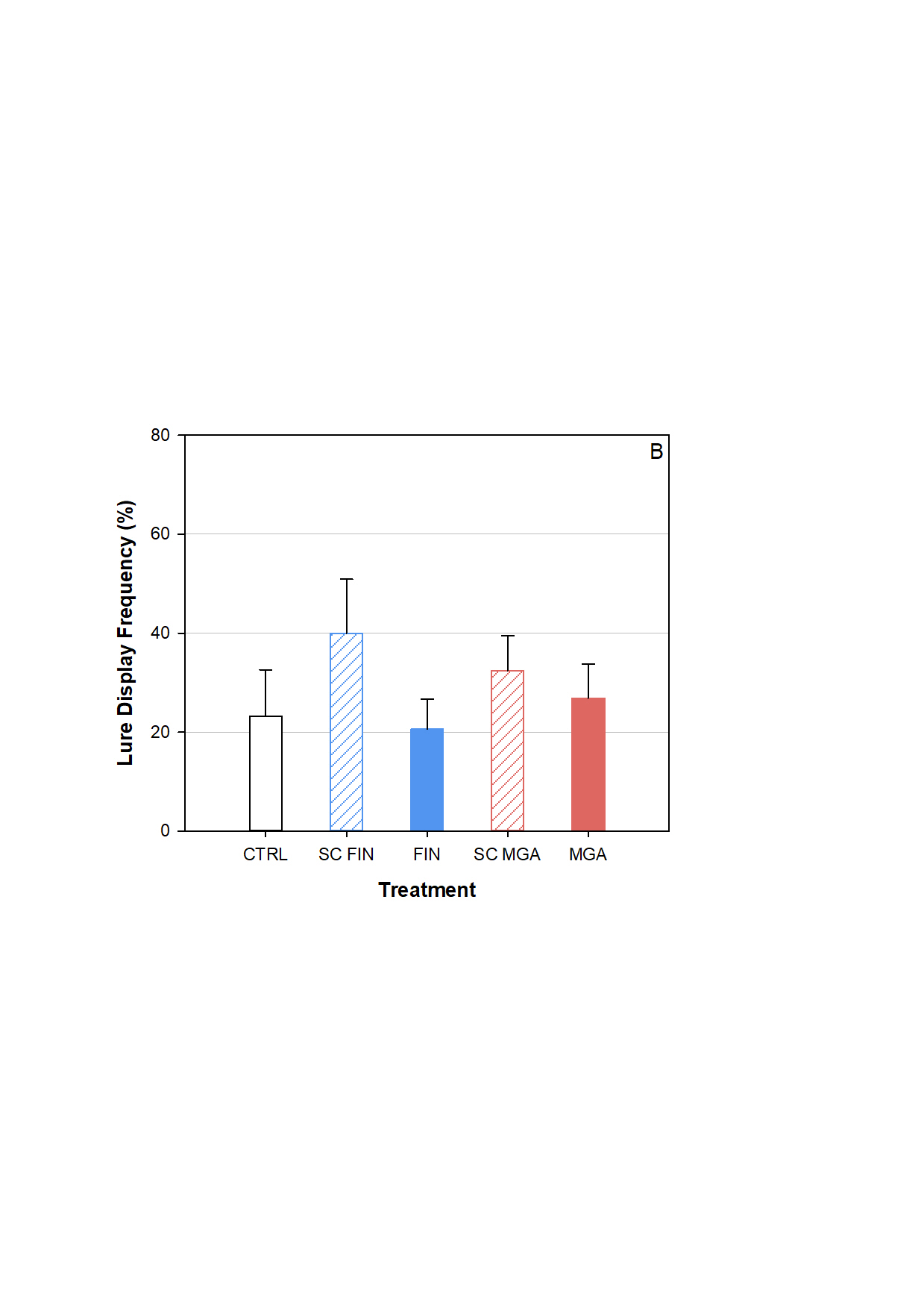


**Figure S2** Filtering frequency (A) and lure display frequency (B) of gravid mussels (*Lampsilis fasciola*) (mean ± standard error of the mean, *n* = 5) monitored three times daily on weekdays (*n* = 29), during a 14 d sediment exposure to 10 mg/kg dw finasteride (FIN) or 100 mg/kg dw melengestrol acetate (MGA). CTRL = negative control, SC = solvent control. No differences between treatments were observed (*p* ≥ 0.32, ANOVA).

# Chemical Analysis

## Sample Preparation and Extraction

Water samples with expected concentrations of finasteride (FIN) and melengestrol acetate (MGA) > 10 ppb were diluted 1:1 with methanol, while water samples with trace levels of FIN and MGA (< 10 ppb) were concentrated using Oasis HLB solid phase extraction (SPE) cartridges (6 cc; Waters, Mississauga, ON). Cartridges were conditioned with 6 mL of methanol, followed by 6 mL of MilliQ water. Samples (400 mL) were passed through the SPE cartridges at a rate of ~ 4 mL/min. Analytes were eluted using 20:80 methanol:acetone (HPLC grade; Fisher Scientific, Whitby, ON). Extracts were reduced to dryness with a gentle stream of high purity nitrogen, reconstituted in 200 μL of 50:50 methanol:water, and stored at -10°C prior to analysis.

Sediment samples were centrifuged (3500 rpm for 4 minutes) to remove pore water, freeze-dried for 24 h and homogenized. Freeze dried sediments (1 g) were extracted in polypropylene centrifuge tubes using 5 mL of methanol and sonicated for 10 min. The extract was then centrifuged (3500 rpm for 4 minutes) and the solvent layer transferred to a clean centrifuge tube using a glass transfer pipette. The samples were extracted three times and the solvent layers (~ 15 mL total) were combined. Sample volume was reduced to 10 mL under a gentle stream of high purity nitrogen. Extracts were stored at -10°C prior to analysis.

## Sample Analysis and Quantitation

Extracts were separated on a Thermo Acclaim RSLC120 C_18_ column (2.2 μm, 4.6 x 150 mm) equipped with a C18 guard cartridge, using a Shimadzu 10A liquid chromatography instrument and a Perkin Elmer 200 Series autosampler paired with an AB Sciex Qtrap 5500 mass spectrometer (Concord, ON). Mobile phases were 20 μM ammonium acetate in MilliQ water (A) and methanol (B). The gradient was held at 5% B for 2 min, increased to 100% B over 1 min and held for 2 min, returned to initial conditions over 1 min and re-equilibrated for 4 min; resulting in a total runtime of 10 min. FIN was observed at a retention time (t_R_) of 4.78 min and MGA was observed at a t_R_ of 4.98 min (Figure S1). The MS was operated using the turbo ion spray source and in positive ion mode. Curtain and ion sources gases 1 and 2 were all ultra-high purity nitrogen (Praxair, Peterborough, ON, Canada) and were all applied at a flow rate of 16, 25 and 25 L/min, respectively. The source temperature was 250°C and the ion spray voltage was -5500 V. Samples were analyzed using multiple reaction monitoring. All compound specific mass spectrometer parameters were determined during infusion (10 μL) of FIN and MGA standards using the instrument’s built-in syringe pump (Table S8).

Prior to analysis, all sample extracts were spiked with internal standards FIN-D_9_ and MGA-D_10_. Samples were quantified using calibration curves that plotted peak area, normalized by the internal standard response versus concentration. All calibration curves standards were matrix matched, calibration standards for water samples were 50:50 methanol:water and calibration standards for sediment samples were made up in pooled extracts from blank sediment. Two precursor ion→ product ion transitions were monitored for each analyte; however, only the most intense transition (Table S8) was used for quantitation.

Method limits of quantitation (MLOQ), defined as the average blank response plus 10 times the standard deviation, were 0.1 and 0.2 μg/L for FIN and MGA, respectively, in water samples and 7 and 6 ng/g (dry weight) for FIN and MGA, respectively, in sediment samples. Prior to analysis, the LC-MS was flushed with methanol. Blanks were analyzed before and after the calibration curve and every ten samples. Replicates were also analyzed every ten samples. Calibration curves were run daily and all R^2^ values were greater than 0.95. Recoveries of samples spiked with FIN and MGA at various concentrations were greater than 80% in both water and sediment samples.

**Table S8** Optimized parameters for the analysis of finasteride and melengestrol acetate on an AB Sciex 5500 Qtrap including precursor and product ions (Q1 and Q3 respectively), declustering potential (DP), collision energy (CE) and collision cell exit potential (CXP). Two precursor ion→ product ion transitions were monitored for each analyte. The most intense transition (listed first for each analyte) was used for quantitation.

| Analyte | Q1 (m/z) | Q3 (m/z) | DP (V) | CE (eV) | CXP (V) |
| --- | --- | --- | --- | --- | --- |
| Finasteride | 373  373 | 305  317 | 91  91 | 41  29 | 20  32 |
| Finasteride-D9 | 382  382 | 314  318 | 91  91 | 41  29 | 20  32 |
| Melengestrol acetate | 397  397 | 279  337 | 86  86 | 29  19 | 22  24 |
| Melengestrol acetate-D10 | 399  399 | 279  339 | 86  86 | 29  19 | 22  24 |


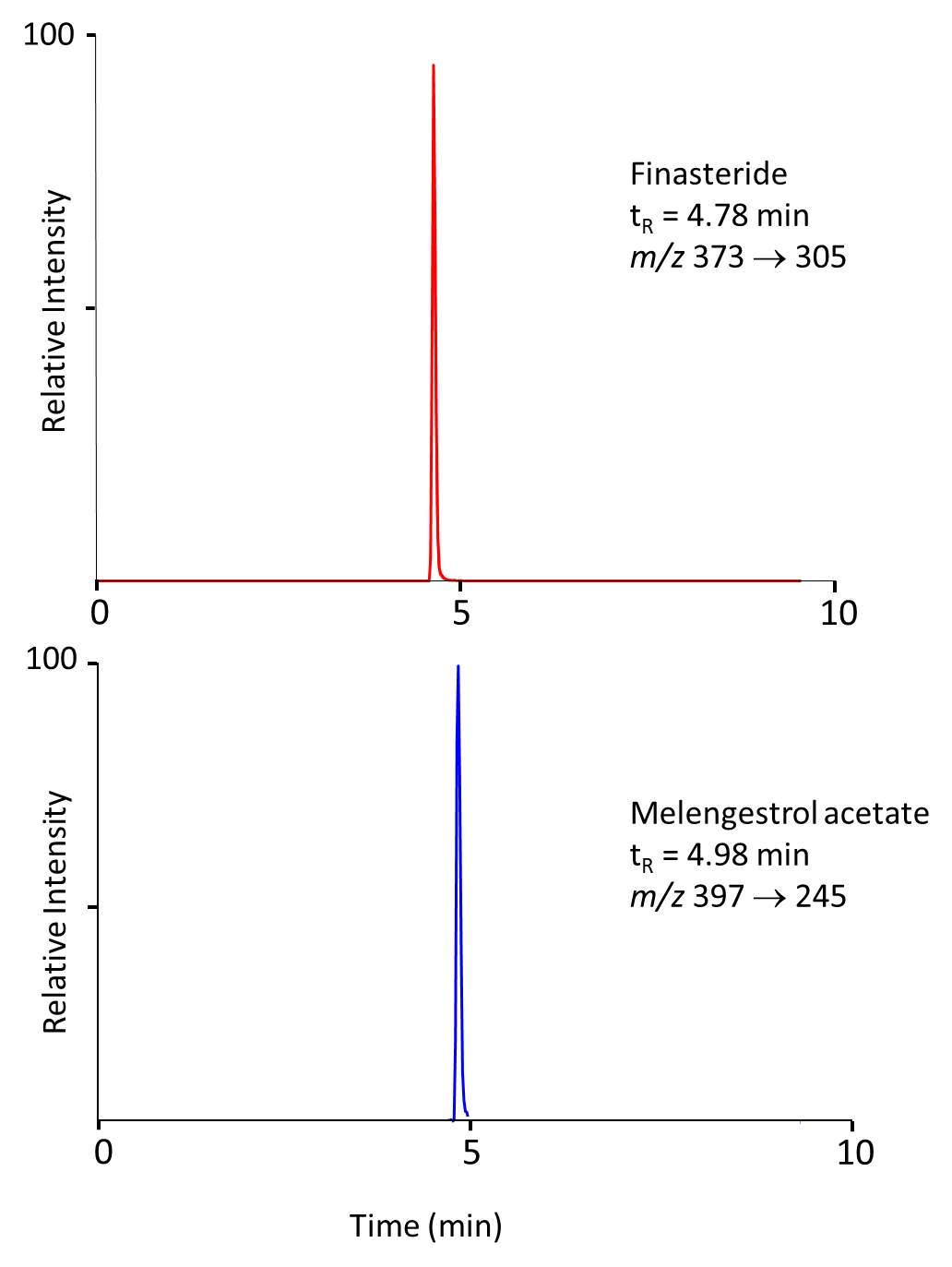


**Figure S3** Sample chromatogram showing separation of finasteride, t_R_ 4.78 min, and melengestrol acetate, t_R_ of 4.98 min.
